# What drives hyperammonemic encephalopathy in AED users: monotherapy risks or polypharmacy perils?

**DOI:** 10.3389/fphar.2025.1477127

**Published:** 2025-06-18

**Authors:** Qing Shan, Lei Wang, Xia Liu, Yan Chen, Bing Li, Yuhang Guo, Qiang Fu, Jinmin Guo

**Affiliations:** ^1^ Department of Clinical Pharmacy, The 960 Hospital, Jinan, Shandong, China; ^2^ Jinan Key Laboratory of Individualised Clinical Drug Safety Monitoring and Pharmacovigilance Research, Jinan, China; ^3^ Department of Orthopedic, The 960 Hospital, Jinan, Shandong, China; ^4^ Department of Clinical Pharmacy, School of Pharmacy, Naval Medical University, Shanghai, China; ^5^ Department of Medical Service, The 960 Hospital, Jinan, Shandong, China

**Keywords:** hyperammonemic encephalopathy, antiepileptic drugs, sodium valproate, perampanel, topiramate, olanzapine, quetiapine

## Abstract

**Background and objectives:**

Hyperammonemic encephalopathy (HE) is a serious side effect linked to sodium valproate (VPA). Recent case studies indicate that newer antiepileptic drugs (AEDs) might also trigger HE, whether used alone or alongside VPA. This study investigated the risk factors of HE linked to 10 AEDs using data from the FDA Adverse Event Reporting System (FAERS), focusing on VPA co-administration effects.

**Methods:**

FAERS reports from the first quarter of 2013 to the third quarter of 2024 were examined for ten frequently prescribed antiepileptic drugs (AEDs): VPA, perampanel (PER), phenytoin (PHT), carbamazepine (CBZ), topiramate (TPM), lamotrigine (LTG), levetiracetam (LEV), oxcarbazepine (OXA), clonazepam (CZP), and zonisamide (ZNS). Hepatic event (HE) signals were evaluated using reporting odds ratios (ROR). A multivariate logistic regression analysis was conducted to assess risk factors (age, gender, indication, drug combinations). Particular attention was given to the effects of VPA in combination with LEV, TPM, olanzapine (OLZ), or quetiapine (QTP) on the risk of HE.

**Results:**

A total of 1,456 HE-related events were identified, with 93.06% of these events linked to AEDs. VPA had the highest association with HE (ROR = 122.14, 95% CI: 110.16–135.41), followed by PER, which was independent of VPA (ROR = 52.62). Eight additional AEDs also indicated positive associations, mainly influenced by VPA (such as TPM and LEV). Identified risk factors for HE included age (with a lower risk observed in minors, OR = 0.61, 95% CI [0.50–0.76]) and clinical indication (with a lower risk in psychiatric disorders, OR = 0.74, 95% CI [0.62–0.89]). The combination of VPA+TPM significantly raised the risk of HE (OR = 3.38, 95% CI [2.25–5.06]) without negatively impacting outcomes. Furthermore, combinations of antipsychotic medications with VPA also indicated an increased risk of HE (OLZ+VPA: OR = 1.65, 95% CI [1.18–2.30], QTP+VPA: OR = 1.95, 95% CI [1.39–2.75]).

**Conclusion:**

This research underscores the possible danger of HE related to AEDs, with a particular focus on the risks tied to VPA and PER when used alone, as well as VPA in conjunction with TPM, OLZ, or QTP. It emphasizes the need to monitor ammonia levels in patients on AEDs, particularly those on polypharmacy.

## Introduction

Hyperammonaemic encephalopathy (HE) is a condition characterized by dysfunction of the central nervous system due to excessively high levels of ammonia (NH_3_) in the blood (hyperammonaemia) (Albrecht et al., 2007). Its etiology is closely linked to disorders of the urea cycle, acute liver dysfunction, and drug toxicity (Vakrinou et al., 2022). Among the pharmacological agents implicated in drug-induced HE, the antiepileptic drug (AED) valproic acid (VPA) has been identrified as a significant contributor. The clinical presentation of HE is diverse, encompassing symptoms such as somnolence, fatigue, exacerbation of seizures, behavioral alterations, insomnia, altered consciousness, unsteady gait, and nystagmus (Schiavo et al., 2023). The emergence of these symptoms typically correlates with the severity of the condition, and without timely intervention, HE can progress to a life-threatening state of coma, underscoring the importance of early detection and diagnosis to enhance patient outcomes.

The association between VPA, a widely used AED, and HE has been has been extensively documented in numerous studies, indicating a significant correlation between the two. The mechanism by which VPA induces HE may involve the inhibition of critical enzymes in the urea cycle, leading to impaired ammonia clearance and astrocyte swelling, as well as disruption of glutamine metabolism (Dabrowska et al., 2018). Research has indicated that factors such as patient age (Vivekanandan and Nayak, 2010) and the concomitant use of hepatic enzyme inducers (e.g., phenobarbital, phenytoin (PHT), and carbamazepine (CBZ)) (Vivekanandan and Nayak, 2010) can elevate the risk of VPA-induced HE. In recent years, the increasing utilization and development of antiepileptic medications have resulted in a rise in reported cases of HE, drawing significant attention from the medical community. For instance, a case involving a 27-year-old male patient with autism, microcephaly, developmental delays, and epilepsy was documented, wherein drug-induced HE was suspected to be associated with the use of perampanel (PER) (Marques et al., 2021). Additionally, there have been reports of HE potentially arising from the use of CBZ or topiramate (TPM) as monotherapy, highlighting the necessity for further investigation into the risks of HE associated with commonly prescribed AEDs (Adams et al., 2009; Tantikittichaikul et al., 2015). In the management of epilepsy and psychiatric disorders, monotherapy is often inadequate, necessitating long-term combination therapy. Case reports indicate that the incidence of HE is frequently linked to the concurrent use of multiple medications. For example, the addition of agents such as zonisamide (ZNS), levetiracetam (LEV), TPM, and quetiapine (QTP) to a regimen that includes VPA has been associated with the onset of HE (Roh et al., 2014; Ilieva et al., 2020; Zhao et al., 2021; Baumgartner et al., 2019). Notably, a significant cohort study comprising 8,372 patients indicated that the concomitant use of TPM and VPA was associated with an approximately ten-times increase in the risk of encephalopathy when compared to treatment with VPA alone (Noh et al., 2013). This observation has prompted scholarly interest in the additional risk posed by the co-administration of VPA with other pharmacological agents, although there remains a paucity of relevant real-world data.

In conclusion, given the potential clinical implications, thorough investigations are warranted to elucidate the relationship between AEDs and HE, as well as the factors that may influence this association. Utilizing data from the FAERS database spanning from Q1 2013 to Q3 2024, this research aims to quantify adverse events related to HE. The research will employ disproportionality analysis to examine adverse events linked to HE, with particular emphasis on the effects of AEDs monotherapy and the combination of sodium valproate (VPA) regimens on the risk of developing HE. Additionally, the investigation will explore other potential contributing factors, biological mechanisms, temporal characteristics, and outcomes associated with ADEs. The objective of this research is to provide a comprehensive understanding of HE-related adverse events associated with AEDs, thereby offering valuable insights for clinical practice.

## Methods

### Data sources

The current investigation relies on the FAERS (FDA Adverse Event Reporting System) database for pharmacovigilance research. This database is recognized as the largest post-marketing pharmacovigilance platform globally, offering extensive data resources for analysis. The study focuses on ten frequently prescribed antiepileptic drugs (AEDs): VPA, PER, PHT, CBZ, TPM, ZNS, LEV, oxcarbazepine (OXA), clonazepam (CZP), and lamotrigine (LTG). These drugs were utilized as keywords to retrieve reported adverse drug events (ADEs) from the FAERS database for the period spanning from 1 January 2013, to 30 September 2024. The analysis included only cases with suspected ADEs. Adverse reactions documented in the FAERS database were coded according to the Preferred Terminology (PT) codes established in the Medical Dictionary for Regulatory Activities (MedDRA). For this study, the PT term for Hyperammonemic Encephalopathy (SOC code: 10067327) was selected based on MedDRA version 24.1 for subsequent analyses.

### Data processing procedures

**FIGURE 1:**
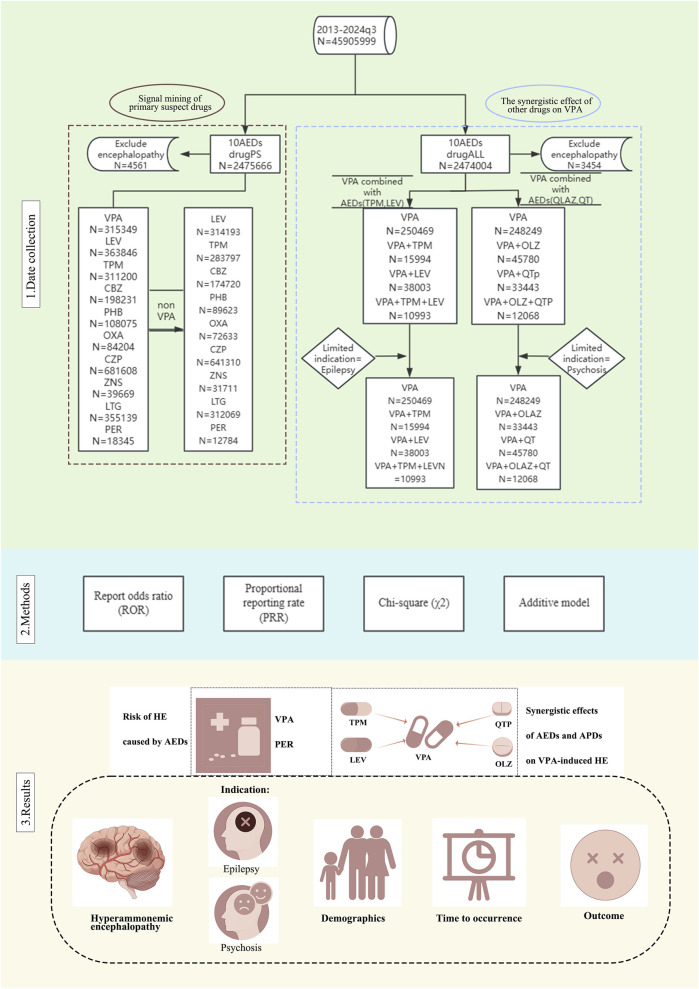
Flow chart of the study design and antiepileptic drug (AED) classification. The flowchart presents a detailed view of the data collection process, analysis methodology and key findings. Abbreviations used include Antiepileptic drugs (AEDs); Sodium valproate (VPA); Topiramate (TPM); Levetiracetam (LEV); Phenytoin (PHT); Carbamazepine (CBZ); Oxcarbazepine (OXA); Perampanel (PER); Zornisamide (ZNS); Clonazepam (CZP); Lamotrigine (LTG); Antipsychotic Drugs (APD); Quetiapine (QTP); Olanzapine (OLZ); Primary suspect drugs (PS).

In processing the ten reports of AEDs obtained from the FAERS database, an initial step involved the removal of duplicate data, as illustrated in Figure 1. Reports were classified as duplicates if they shared identical values across the domains of gender, age, country, date of event, adverse reaction, drug, and indication. The remaining reports underwent further screening based on specific criteria to exclude reports of psychiatric adverse events potentially attributable to other factors. These criteria included: (1) the presence of terms such as ‘septic encephalopathy,’ ‘allergic encephalopathy,’ ‘hepatic encephalopathy,’ or ‘autoimmune encephalopathy’ as therapeutic indications for cases related to encephalopathy; and (2) the presence of concomitant medications that may have been prescribed for the treatment of epileptic disorders, such as VPA. It is important to note that while the influence of these non-ADE factors can be mitigated, it cannot be entirely eliminated. Additionally, treatment strategies for ADEs were subjected to further screening, retaining reports of both ADEs monotherapy and combination therapy, specifically considering combinations of VPA with LEV, TPM, olanzapine (OLZ), and QTP. Following the deduplication and screening processes, the overall reports of adverse reactions for patients treated with ADEs in the FAERS database were compiled for further analysis (N = 2,475,666).

### Single-drug signal mining and risk factor analysis

This study employs the ratio of reports (ROR) methodology, proportional reporting rate (PRR), and chi-square (χ^2^) to identify signals of Hyperammonemic Encephalopathy (HE) within reports of the ten ADEs. The ROR is calculated by comparing the ratio of the target adverse event to all other events for a specific drug against the ratio of the target adverse event to all other events for a non-specific drug. Positive disproportionation signals were defined as a lower bound of a ≥3, 95% CI > 1, PRR ≥ 2, χ^2^ ≥ 4, and *P* < 0.05 (Borrelli et al., 2018). The formula for calculating the ROR is detailed in Supplementary Table S3. Concurrently, relevant patient information, including gender, age, and indication, was extracted and analyzed through multifactorial logistic regression to identify risk factors associated with drug-related HE. The indications pertinent to this study, encompassing the comprehensive classification and inclusion criteria for psychiatric disorders and epilepsy, have been explicitly delineated in Supplementary Table S1.

### Joint drug signal mining and risk factor analysis

The present study examines the effects of VPA in conjunction with commonly used antiepileptic drugs and antipsychotics (LEV, TPM, OLZ, QTP) on HE, utilizing signal mining and risk factor analysis of co-administration. Disproportionality analyses were conducted using reports of VPA as a single agent as the reference group, assessing the potential association between VPA combinations and pharmacological HE through ROR. Additionally, the combination data were re-evaluated using an additive model, positing that drug-related hazards may amplify the potential risk, with the relevant formulas provided in Supplementary Tables S4,S5 (Noguchi et al., 2020). Multifactorial logistic regression analyses were also performed. To mitigate the potential influence of indication on HE, the analysis of VPA in combination with LEV and TPM was limited to patients diagnosed with epilepsy, while the analysis of VPA in combination with OLZ and QTP was restricted to patients with psychosis.

## Results

### Signal and risk factor analysis of monotherapy for 10 AEDs

The current investigation examined the reporting rate of HE reporting among patients receiving treatment with ten AEDs as recorded in FAERS from the first quarter of 2013 to the third quarter of 2024. A total of 1,456 HE were documented within the dataset, of which 1,355 (93.06%) were associated with ADEs, affecting 1,264 patients. A gender-based analysis indicated a slight increase in reporting among female patients (48.81%) in comparison to male patients (43.12%). Additionally, the age distribution revealed a significant concentration of cases within the 18–64 age demographic (70.73%), while a smaller proportion was observed among minors (14.87%) and the elderly (4.83%). Concerning adverse outcomes, it is significant to note that 99.68% of patients experienced at least one serious adverse outcome, with 5.06% resulting in death (DE) and 15.27% classified as life-threatening (LT). In terms of geographic distribution, the majority of reported cases originated from countries in the Americas (35.60%) and Europe (28.72%). Further details are available in Supplementary Tables S6,S7.

**FIGURE 2:**
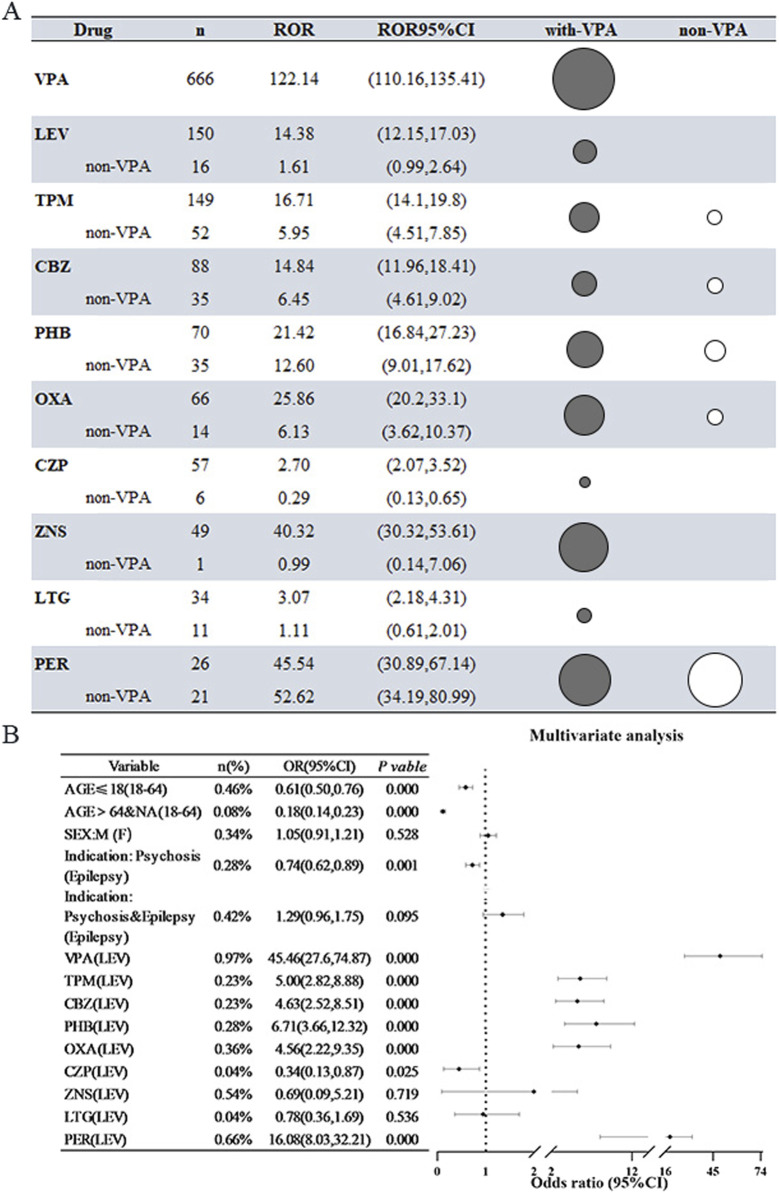
Risk of Hyperammonemic Encephalopathy (HE) Associated with 10 Antiepileptic Drugs (AEDs) **(A)** The reporting odds ratio (ROR) for all antiepileptic drugs (AEDs) and for AEDs after removing cases related to valproic acid (VPA). In this instance, the black bubble plots indicate the ROR magnitude for all AEDs, while the white bubbles show the ROR values after excluding cases in combination with VPA. **(B)** Findings from logistic regression analyses concerning AEDs that result in hepatotoxicity (HE) after excluding cases associated with VPA. Abbreviations used include Sodium valproate (VPA); Topiramate (TPM); Levetiracetam (LEV); Phenytoin (PHT); Carbamazepine (CBZ); Oxcarbazepine (OXA); Perampanel (PER); Zornisamide (ZNS); Clonazepam (CZP); Lamotrigine (LTG); Reporting Odds Ratio (ROR); 95% Confidence Interval (95% CI); Odds Ratio (OR).

A comprehensive analysis was conducted on ten AEDs utilizing both the ROR method and the PRR method, with all drugs exhibiting significant signals. Notably, the association between VPA and the incidence of HE was particularly pronounced, yielding an ROR value of 122.14 (95% CI: 110.16–135.41), which indicates a robust correlation with HE. Furthermore, a substantial proportion of cases involving the other nine AEDs were found to be in conjunction with VPA. To more accurately evaluate the independent risk associated with the remaining drugs, we repeated the signal analysis after excluding VPA. The findings revealed a marked reduction in signal intensity for the majority of the AEDs, with only five maintaining a positive signal. For instance, the signal intensity for LEV diminished from 14.38 to 1.61, while that of TPM decreased from 16.72 to 5.95. This indicates that the vigilance signals associated with these pharmacological agents were significantly impacted by their interaction with VPA. It is important to highlight that the signal associated with PER exhibited a considerable level of stability and significance, remaining unaffected by VPA. The overall signal strength of PER ranked just below that of VPA, suggesting a robust independent correlation with hypertension (refer to Figure 2A).

A thorough investigation into the risk factors associated with HE related to AEDs was performed, incorporating adjustments for potential confounding variables such as age, sex, and clinical indication. The results indicated that the likelihood of developing AED-associated HE was 0.61 times lower in patients under the age of 18 compared to those aged 18–64 years (OR = 0.61, 95% CI [0.50–0.76], p < 0.001). When examining various disease types, individuals with psychiatric disorders were significantly less likely to report HE, with patients diagnosed with epilepsy serving as the reference group (OR = 0.74, 95% CI [0.62–0.89], P = 0.001). Regarding pharmacological treatments, LEV was utilized as a control, while several other medications—including VPA, TPM, CBZ, PHT, OXA, and PER—were found to significantly elevate the risk of HE, with ORs ranging from 4.56 to 45.46. Notably, with the exception of VPA, the risk associated with PER was particularly significant (OR = 16.08, 95% CI [8.03–32.21], P < 0.001), indicating that this newer AED warrants careful consideration in clinical practice due to its heightened risk of HE (refer to Figure 2B). The relevant multifactorial analyses that did not exclude the effects of VPA are shown in Supplementary Figure S1.

### Joint drug signal mining and risk factor analysis of VPA in combination with TPM or LEV

The aforementioned study identified TPM and LEV as the most frequently prescribed AEDs alongside VPA. Among the 641 cases of HE analyzed, 457 patients were treated exclusively with VPA, 56 patients received a combination of TPM+VPA, 93 patients were administered VPA in conjunction with LEV, and 35 patients were treated with all three medications concurrently. The patient demographic predominantly consisted of individuals aged between 18 and 64 years, accounting for 53.57%–77.42% of the sample, while the proportion of minors ranged from 8.57% to 21.34%. Additionally, the distribution of male and female patients was relatively balanced, with male patients comprising 42.90% and female patients 49.30%, revealing no statistically significant differences (refer to Supplementary Tables S8,S9).

**FIGURE 3:**
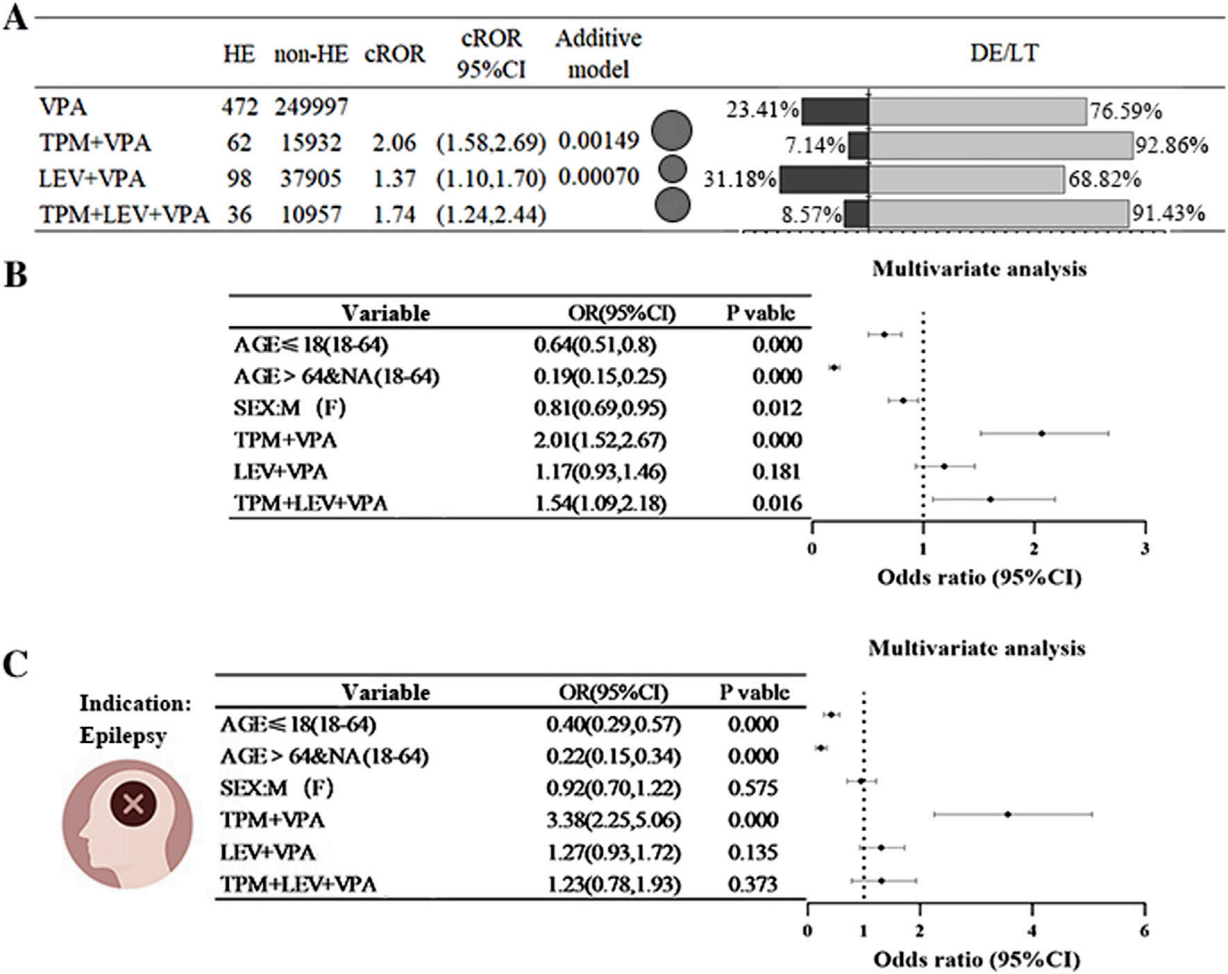
Risk of Hyperammonaemic Encephalopathy Resulting from the Combination of Sodium Valproate with Antiepileptic Drugs (Topiramate or Levetiracetam) **(A)** A comparison of the reported ratio of values (ROR) and the percentage of outcomes related to hyperammonaemic encephalopathy (HE), death (DE), or life-threatening (LT) situations when sodium valproate is administered alongside topiramate (TPM) or levetiracetam (LEV). The black bubbles represent ROR values; in the dual bar graphs, the black section shows the percentage of death (DE) or life-threatening (LT) outcomes, while the grey section represents the percentage of other outcomes. **(B)** Findings from a multifactorial analysis regarding the incidence of HE when sodium valproate is used with topiramate (TPM) or levetiracetam (LEV). **(C)** In combination, outcomes of a multifactorial analysis of HE occurrences in epilepsy patients treated with sodium valproate, topiramate (TPM), or levetiracetam (LEV) were assessed. Abbreviations used include Sodium valproate (VPA); Topiramate (TPM); Levetiracetam (LEV); Death (DE); Life-threatening (LT); Reporting Odds Ratio (ROR); 95% Confidence Interval (95% CI); Odds Ratio (OR).

The combination regimens of TPM+VPA, LEV+VPA, and TPM+LEV+VPA exhibited an increased risk relative to treatment with VPA alone, as indicated by ROR values of 2.06, 1.37, and 1.74, respectively. Further validation through additive modeling corroborated the existence of signals for both drug combination regimens. The incidence of serious adverse outcomes, characterized as either DE or LT events, varied between 7% and 28% across the four treatment regimens (refer to Figure 3A). To further clarify the impact of these combinations on HE, multifactorial analyses were conducted, controlling for age and sex as covariates. The combination of TPM+VPA was found to significantly elevate the risk of HE associated with VPA, yielding a ratio of 2.01 (OR = 2.01, 95% CI [1.52, 2.67]), while the triple-drug combination of TPM+LEV+VPA also increased the risk of HE (OR = 1.54, 95% CI [1.09, 2.18]). The LEV+VPA regimen also demonstrated some degree of risk, though this did not reach statistical significance (OR = 1.17, 95% CI [0.93, 1.45], P = 0.181) (see Figure 3B). When the analysis focused exclusively on patients diagnosed with epilepsy, the association between the combination of TPM+VPA exhibited a markedly increased signal compared to the analysis of the entire population (OR = 3.38, 95% CI: [2.25, 5.06]). Similarly, LEV did not achieve statistical significance, which aligns with the findings from multifactorial analyses conducted on the unrestricted population (see Figure 3C).

### Joint drug signal mining and risk factor analysis of VPA in combination with OLZ or QTP

In our analyses of monotherapy, it was observed that patients with psychiatric disorder indications constituted roughly one-fifth of the overall population. Although the incidence of HE within this subgroup was relatively low, it prompted us to consider the potential interactions between antiepileptic medications and antipsychotic agents. Consequently, we opted to conduct a more in-depth examination of the effects of OLZ and QTP, two antipsychotic medications frequently administered in conjunction with VPA in clinical settings. In a study involving 639 HE patients, treatment regimens varied among the participants: 460 received only VPA, 100 were administered a combination of QTP+VPA, 61 were treated with OLZ in conjunction with VPA, and 35 patients were prescribed all three medications. The age distribution of the patients predominantly ranged from 18 to 64 years, comprising 71.21% of the cohort, while minors constituted 15.34%. Furthermore, the gender distribution was relatively balanced, with 43.04% of the patients being male and 49.14% female, indicating no statistically significant difference (refer to Supplementary Tables S10,S11 for further details).

**FIGURE 4:**
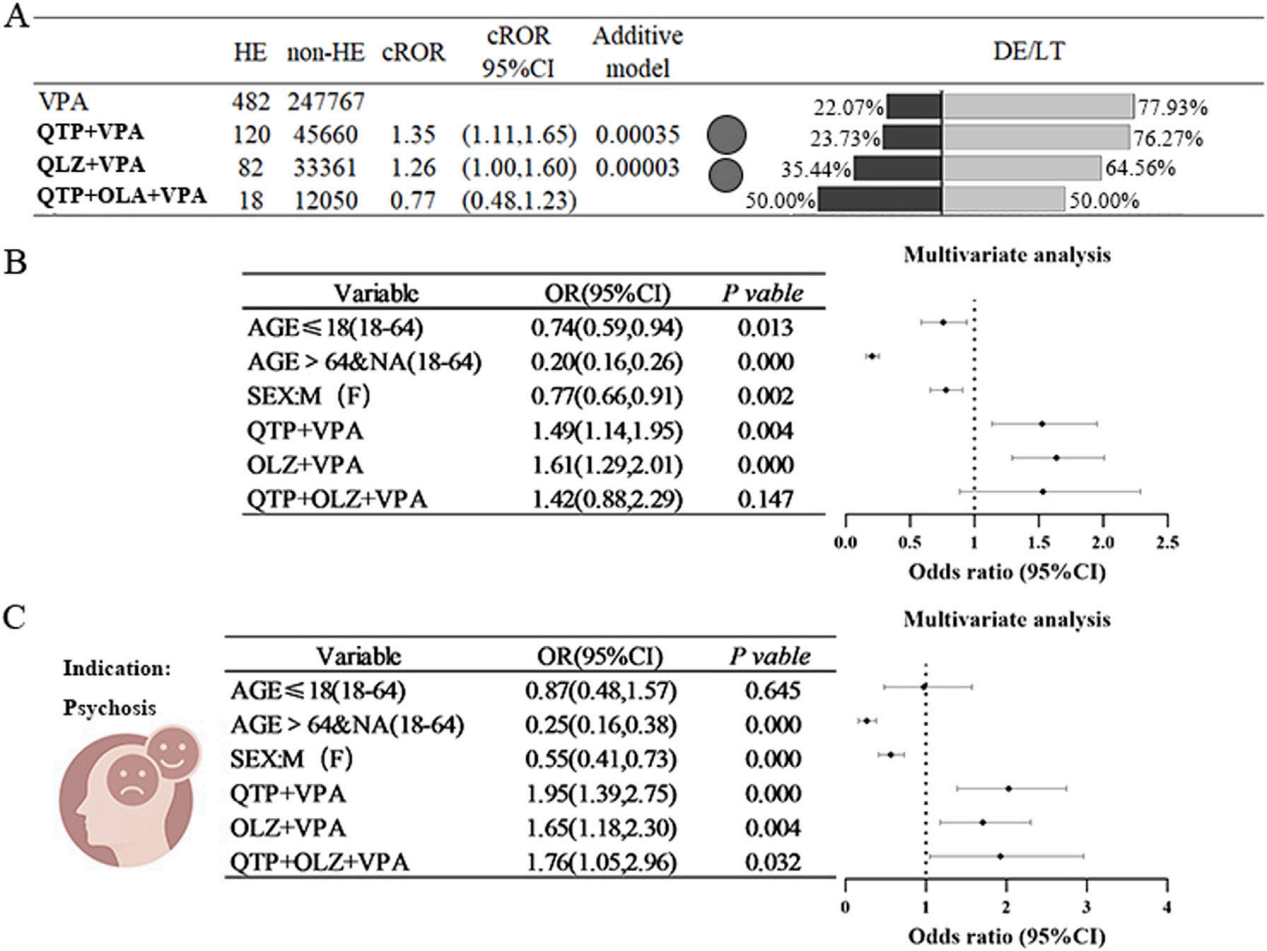
Risk of Hyperammonaemic Encephalopathy due to the combination of sodium valproate and antipsychotics (quetiapine or olanzapine) **(A)** It compares the ratio of reported values (ROR) for sodium valproate combined with quetiapine (QTP) or olanzapine (OLZ) against the percentage of outcomes related to hyperaemic encephalopathy (HE), death (DE), or life-threatening situations (LT). The black bubbles represent ROR values; in the double bar graph, the black section shows the percentage of death (DE) or life-threatening (LT) outcomes, while the grey section represents other outcomes. **(B)** Displays the results of a multifactorial analysis regarding the incidence of HE when valproate is used with quetiapine (QTP) or olanzapine (OLZ). **(C)** Shows the results of a multifactorial analysis of HE incidence in epilepsy patients treated with valproate in combination with quetiapine (QTP) or olanzapine (OLZ). Abbreviations used include Sodium valproate (VPA), Quetiapine (QTP), Olanzapine (OLZ), Death (DE), Life-threatening (LT), Reporting Odds Ratio (ROR); 95% Confidence Interval (95% CI); Odds Ratio (OR).

Preliminary results indicated that both the OLZ+VPA combination regimen and the QUE+VPA combination regimen demonstrated positive signals, as evidenced by the ROR (QUE+VPA:ROR = 1.35; OLZ+VPA:ROR = 1.26), whereas the three-drug combination did not yield similar results (only 18 cases). Further validation through additive modeling confirmed significant signals for both two-drug combination regimens (refer to Figure 4A). In a subsequent multifactorial analysis that were adjusted for age and sex, the combination of OLZ+VPA and QUE+VPA was associated with a 1.49 to 1.61 times increase in the risk of HE compared to VPA administered alone (refer to Figure 4B). When the analysis was limited to individuals diagnosed with psychiatric disorders, the results indicated that the combinations of OLZ+VPA and QUE+VPA elevated the risk of HE by 1.65 times (OR = 1.65, 95% CI [1.18, 2.30]) and 1.95 times (OR = 1.95, 95% CI [1.39, 2.75]), respectively. These results suggest a relatively consistent risk signal in this specific population compared to the broader cohort. Furthermore, the combination of OLZ+QUE+VPA resulted in a lower incidence of HE, with only 18 cases reported. No statistically significant difference was observed in the pre-post multifactorial analysis of the entire population; however, a statistically significant difference was noted within the restricted psychiatric population (refer to Figure 4C).

### Utilization of VPA in pediatric patients and related risks

**FIGURE 5:**
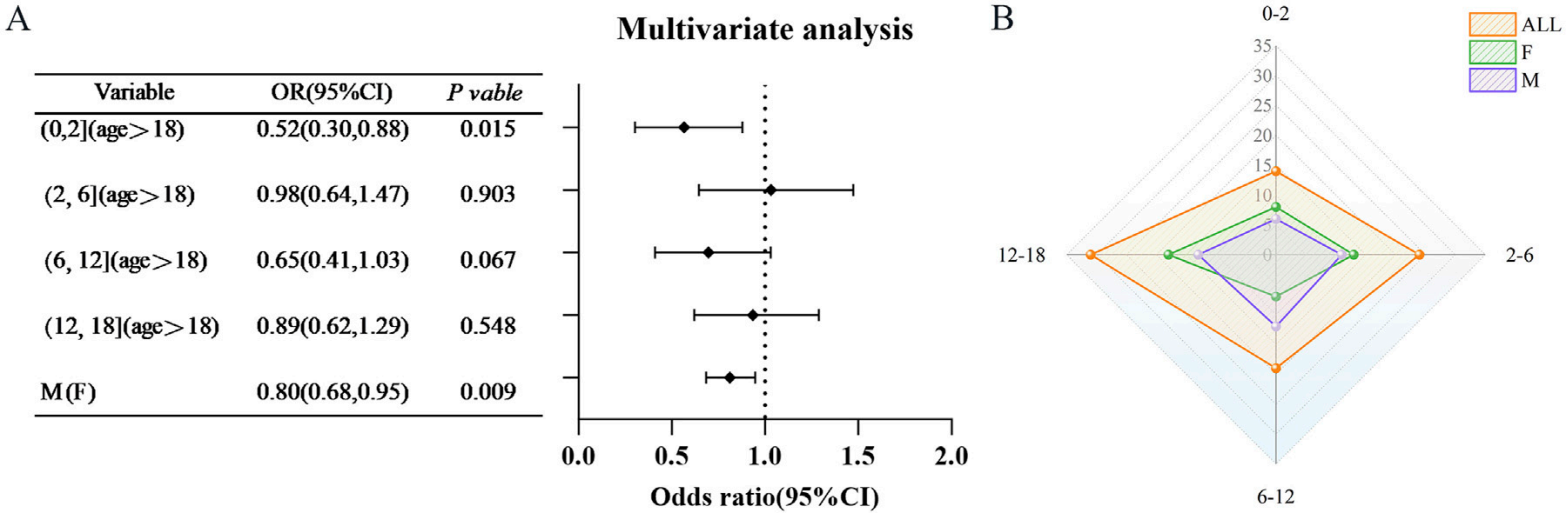
Age-specific risk of hyperammonaemic encephalopathy (VHE) caused by sodium valproate in children **(A)** Findings from multifactorial analyses of VHE occurrences in children across various age categories. **(B)** Distribution of VHE cases among children in each age category. Red represents the overall population proportion, green indicates the proportion of females, and purple shows the proportion of males. Abbreviations used include Reporting Odds Ratio (ROR); 95% Confidence Interval (95% CI); Odds Ratio (OR).

The findings indicate that the underage population exhibited certain protective effects in both monotherapy and multidrug combination therapy. In light of these results, the current study aims to further investigate the implications of underage status across various age groups, utilizing VPA) as a case study. A total of 88 instances of underage cases associated with VPA-related HE) were documented, categorized by age as follows: 14 cases in the 0–2 years age group, 24 cases in the 2–6 years age group, 19 cases in the 6–12 years age group, and 31 cases in the 12–18 years age group. After adjusting for age and sex, the protective effect of VPA was predominantly observed in the 0–2 years age group (OR = 0.52, 95% CI [0.30, 0.88]) (refer to Figure 5).

### Examination of time-based outcomes for VPA and TPM

**FIGURE 6:**
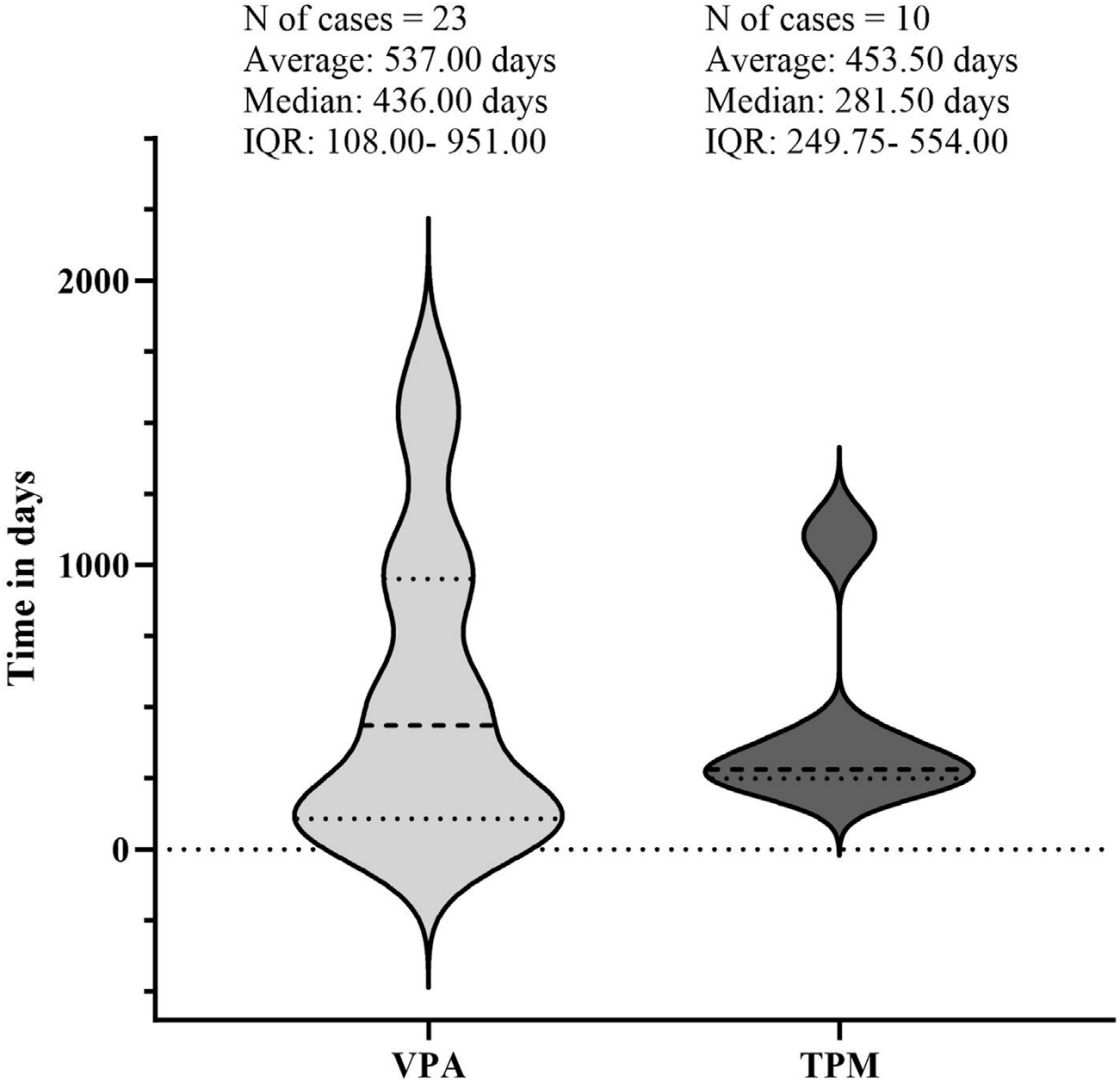
Illustrates the timing of hyperammonaemic encephalopathy (HE) onset following exposure to sodium valproate (VPA) or topiramate (TPM). The lighter regions represent the timeline from VPA exposure to the onset of HE, while the darker regions show the timeline from TPM exposure to HE onset. Abbreviations used include Interquartile Range (IQR).

The temporal occurrence of HE was examined in this study. Following the exclusion of reports lacking temporal data, a total of 33 valid reports were ultimately included in the analysis. The median duration of the 23 HE events associated with VPA was found to be 436 [interquartile range (IQR):108–951] days. The duration of HE varied from 6 months to 3 years post-initiation of treatment, with a predominant occurrence approximately one and a half years after the commencement of therapy. In contrast, the median time to HE events related to TPM was recorded at 281 [IQR: 249.75–554] days. Due to insufficient data, further analysis of the time to HE events for other AEDs was not feasible (refer to Figure 6).

## Discussions

To the best of our knowledge, this study represents the inaugural pharmacoepidemiological investigation utilizing the FAERS database to assess the risk of HE associated with AEDs and the compounded risk of HE when VPA is administered in conjunction with AEDs and antipsychotics. As the preeminent open access database for post-marketing pharmacovigilance, the FAERS database provides a significant advantage in identifying potential red flags for infrequent adverse reactions, including HE (Villa-Zapata et al., 2022). Within this database, HE, characterized as a rare adverse reaction with a low incidence rate, was linked to antiepileptic drugs (AEDs) in 93.06% of the 1,456 reported cases. In alignment with anticipated outcomes, VPA demonstrated the strongest correlation with hepatotoxicity reports. Conversely, it was surprising to observe that the PER signal for the novel AEDs was notably robust and remained unaffected by VPA. Furthermore, the analysis revealed that VPA, when used in combination with other AEDs (specifically TPM and LEV) or with antipsychotics (OLZ and QUE), significantly heightened the risk of HE.

HE, a recognized adverse effect of VPA and its derivatives, is well known in clinical settings. Consistent with expectations, this study confirmed that VPA is associated with a markedly increased risk of HE (ROR = 122.14), thereby reinforcing the hypothesis of a robust correlation between VPA and HE, thereby affirming the reliability of the FAERS database. While HE was infrequently linked with other AEDs, the signal intensity of the majority of AEDs diminished both prior to and following the exclusion of the influence of VPA from the analytical assessment. In contrast, the signal associated with PER exhibited a notable degree of stability (45.54 vs. 52.62). The Case reports on PER-induced HE is sparse, with only a single clinical case documented. This case involved a 27-year-old male patient with epilepsy who had been a long-term user of TPM and was diagnosed with pharmacological HE following the onset of syncope and other symptoms 1 month after the introduction of PER (Marques et al., 2021). The patient’s syncopal episodes improved subsequent to a reduction in the PER dosage, which the authors propose may be associated with the administration of PER. The FAERS database provides valuable supplementary real-world data, revealing a total of 26 reported cases of PER-induced HE. This figure is noteworthy for such a rare condition, indicating that this relatively novel AED may carry a risk for HE through mechanisms that remain to be fully understood. Furthermore, other AEDs exhibited a weak positive correlation with HE, particularly in conjunction with VPA, a finding that has been corroborated in the majority of case reports.

Combination treatment regimens are frequently employed in the management of epilepsy. Prior research, including several case reports, has indicated that patients may exhibit encephalopathic symptoms following the introduction of TPM to a regimen that includes VPA (Vivekanandan and Nayak, 2010; Zhao et al., 2021; Deutsch et al., 2009; Raru and Zeid, 2018). Notably, the abrupt cessation of VPA (without discontinuing TPM) has been associated with a rapid resolution of encephalopathy symptoms, suggesting a potential causal relationship (Gomez-Ibanez et al., 2011; Twilla and Pierce, 2014). The potential adverse effects associated with VPA continue to be our foremost concern, and the supplementary risks associated with concomitant medication for VPA are equally significant. A single-center cohort study revealed that the use of TPM significantly increased the risk of HE episodes, nearly tenfold, among 8,372 hospitalized patients receiving VPA, with 11 documented cases of drug-related VHE (Noh et al., 2013). In our investigation, the risk of VHE was found to be 2.012 times greater when TPM was used in conjunction with VPA compared to VPA alone. Our research aligns with this observation, indicating that the combination of TPM and VPA elevates the risk of HE. Nevertheless, the extent of this increase varied across the data. It is crucial to acknowledge that the fundamental differences in study design, data sources, and metrics between the two articles render them non-comparable; the former focuses on reporting incidence, while our study is limited to demonstrating a reported rate. While there was a trend indicating an increased risk of VHE episodes with the combination of LEV and valproic acid, this did not achieve statistical significance. The literature contains limited reports on the association between LEV and VHE, with only two documented cases of LEV-induced VHE and a notable absence of relevant clinical trials (Roh et al., 2014; Raru and Zeid, 2018). Typically, the severity of HE ranges from mild to moderate, although it can lead to severe complications and mortality (Izadi et al., 2018). Our study indicated a relatively favorable prognosis for HE, with most cases necessitating hospitalization, aligning with findings from prior studies. In particular, HE deaths were observed in 23.41% of cases involving VPA monotherapy, whereas the incidence was 7.14% for the combination therapy of TPM and VPA, and 31.18% for the combination therapy of LEV and VPA. This suggests that while TPM or LEV may facilitate the onset of VHE, they do not appear to influence the severity or prognosis of the condition. This observation is consistent with cohort studies and case reports indicating that most patients recover following the discontinuation of valproic acid, with no significant prognostic differences between users of TPM+VPA and those on VPA monotherapy. VPA is utilized not only for epilepsy treatment but also as a mood stabilizer in bipolar disorder and schizophrenia. VPA-associated HE has been documented in both neurology and emergency medicine literature, though there are few case reports pertaining to its psychiatric applications (Settle, 1995; Chopra et al., 2012). Our research indicated that 29.5% of patients who experienced HE had a prior history of psychiatric disorders. Furthermore, individuals with psychiatric conditions were less likely to report instances of HE in comparison to those with epilepsy, a finding that aligns with the current body of literature. Additionally, the combination of valproic acid with antipsychotics was examined, revealing that co-administration with OLZ (ROR = 1.35) or QTP (ROR = 1.35) presents an additional risk. The existing case reports indicate the use of a combination of OLZ and QTP with VPA; however, the authors did not propose that this combination resulted in an elevated risk of HE (Dawson et al., 2016). This represents a limitation of the case reports in substantiating any additional risk, thereby underscoring the importance of the analyses conducted in this study utilizing the database.

In conducting our pharmacovigilance study, we utilized an open database of spontaneous reports for a comprehensive analysis of drug safety. Our findings were consistent with real-world reports, providing a robust empirical foundation for our conclusions. Furthermore, our study confirmed that certain adverse drug reactions are indeed linked to the risk of HE, with the underlying pharmacological mechanisms being complex and multifaceted. VPA, a branched-chain short-chain fatty acid, is primarily metabolized through mitochondrial β-oxidation in the liver, a process that requires carnitine for the transport of VPA into mitochondria. Prolonged use, high doses, or overdose of VPA can deplete carnitine levels, leading to elevated blood ammonia levels (Vazquez et al., 2014). Although the precise mechanism by which VPA induces hyperammonaemia is not fully understood, it is widely accepted that VPA disrupts normal ammonia metabolism by directly inhibiting carbamoylphosphate synthetase, a critical enzyme in the urea cycle, and indirectly depleting carnitine. This disruption results in increased ammonia production in the kidneys and diminished liver capacity for ammonia metabolism, culminating in elevated ammonia levels and various toxic effects (Schiavo et al., 2023; Safdar and Ismail, 2023; Shnayder et al., 2023). Additionally, elevated ammonia levels may be exacerbated by drug-drug interactions. In this research, we examined the risk of HE associated with the use of VPA in conjunction with other medications. Given the relatively low incidence of HE linked to VPA in real-world settings, this pharmacovigilance study has yielded novel findings that indicate the necessity for heightened clinical vigilance regarding the co-administration of VPA with other drugs. The potential mechanisms underlying these interactions are elaborated upon in the following sections. The combination of TPM and LEV with VPA has been shown to enhance the unbound (biologically active) fraction of VPA, potentially leading to hyperammonaemia (Meng et al., 2022). However, this hypothesis remains debated, as studies have not consistently demonstrated a clear correlation between daily doses and serum concentrations of valproic acid and the occurrence and severity of drug-related HE (Woo et al., 2020). TPM has been shown to worsen the development of hyperammonaemia and VPA+TPM encephalopathy by depleting L-carnitine and inhibiting carbonic anhydrase and brain glutamine synthetase (Noh et al., 2013). LEV is primarily excreted via the kidneys, and impaired renal function can significantly affect its metabolism (Raru and Zeid, 2018). In patients with renal failure, the clearance of LEV is markedly reduced, which may enhance its toxicity. For instance, a patient with chronic kidney disease developed myoclonic encephalopathy after treatment with LEV, which was hypothesized to result from the drug’s accumulation in the body (Vulliemoz et al., 2009). Furthermore, it has been demonstrated that medications such as PHT, phenobarbital, and CBZ can influence the activity of carbamoyl phosphate synthase (CPS-1) (Farooq et al., 2017). This study investigates the impact of co-administration on the risk of HE associated with VPA. Prior research has indicated that the concomitant use of risperidone with VPA may influence VPA metabolism through its binding to albumin, potentially resulting in elevated blood ammonia levels and an increased risk of HE (Rodrigues-Silva et al., 2013). However, our analysis was unable to corroborate this finding due to a limited number of reports involving risperidone in our database. Conversely, we identified a trend suggesting that two other antipsychotics, OLZ and QTP, may also be associated with an increased risk of HE when administered alongside VPA. This observation opens new avenues for further investigation, even though existing clinical studies have yet to elucidate the precise mechanisms underlying these interactions.

A protective effect on minors has been identified in both monotherapy and multidrug combination analyses. Subsequent investigations, particularly those utilizing valproic acid (VPA) as a case study, indicate that this protective effect is most pronounced within the 0–2 years age demographic. However, it is important to recognize that the limited number of studies focusing on this specific age group constrains the generalizability of these findings. Furthermore, the existing literature concerning minors is inconsistent, with certain studies positing that adolescents may actually represent a risk factor for HE (Vivekanandan and Nayak, 2010). Consequently, there is an urgent need for research involving larger sample sizes to validate these findings within this population. Additionally, the time interval between the onset of HE exhibited considerable variability, ranging from a few days to several years. This observation aligns with previous studies that have established no correlation between daily VPA administration and the incidence or severity of HE (Chopra et al., 2012; Woo et al., 2020). This underscores the necessity for ongoing vigilance regarding the potential for delayed HE in patients undergoing long-term treatment with such pharmacological agents.

We acknowledge that this study had some drawbacks. First, the voluntary nature of FAERS reporting inevitably results in reporting and notoriety bias, as well as missing information, such as comorbidity and drug combination, which have been confirmed as crucial factors in severe outcomes (Villa-Zapata et al., 2022; Meng et al., 2022). Second, it is essential to note that the findings from FAERS should not be automatically interpreted as a causal relationship between drugs and clinical events but rather as an indication of an association, serving as a significant starting point for further research (Meng et al., 2022). Third, the lack of comprehensive drug data prevented the analysis of VHE incidence. The absence of population data on administered AEDs hindered the determination of HE incidence. Fourthly, interactions with other commonly prescribed drugs may be overlooked because the analyses focus on specific drug combinations. Although there are certain constraints, the results of this research suggest possible safety concerns in relation to the emergence of HE when utilizing AEDs. Although uncommon, this potentially fatal adverse response can serve as a guide for medical professionals using AEDs.

## Conclusion

In summary, this research investigated the association between HE and commonly prescribed AEDs, with a particular emphasis on their use as monotherapy and in conjunction with VPA. A notable correlation was identified between VPA and the occurrence of HE. Furthermore, this study presents novel evidence indicating that PER, a recently introduced AEDs, is significantly linked to HE, a finding that warrants further validation through additional clinical studies. Utilizing the FAERS database for analysis, we also discovered that certain drug combinations, particularly the pairing of VPA with TMP, LEV, or OLZ, markedly heightened the risk of HE. These findings underscore the importance of monitoring blood ammonia levels in patients undergoing combination therapy. Most of the reported cases occurred within the first year of drug use. These findings advocate for enhanced pharmacovigilance and the development of targeted therapeutic strategies aimed at mitigating the risk of HE in patients undergoing treatment with AEDs. Additional research is imperative to investigate the underlying mechanisms and to optimize treatment modalities for individuals with epilepsy and related conditions.

## Data Availability

The datasets presented in this study can be found in online repositories. The names of the repository/repositories and accession number(s) can be found below: https://www.fda.gov/drugs/drug-approvals-and-databases/fda-adverse-event-reporting-system-faers.
