# Supplementary material for: What drives hyperammonemic encephalopathy in AED users: monotherapy risks or polypharmacy perils?
Source: Front Pharmacol. 2025 Jun 18;16:1477127. doi: 10.3389/fphar.2025.1477127 (PMC12213827; doi:10.3389/fphar.2025.1477127)
Supplement: Supplementary file 1 [file Supplementaryfile1.docx]

Supplementary Material

# Supplementary Figures and Tables

## Supplementary Figures

Figure1 Risk for hyperammonemic encephalopathy (HE) associated with 10 antiepileptic drugs (AEDs) (**VPA not excluded)**


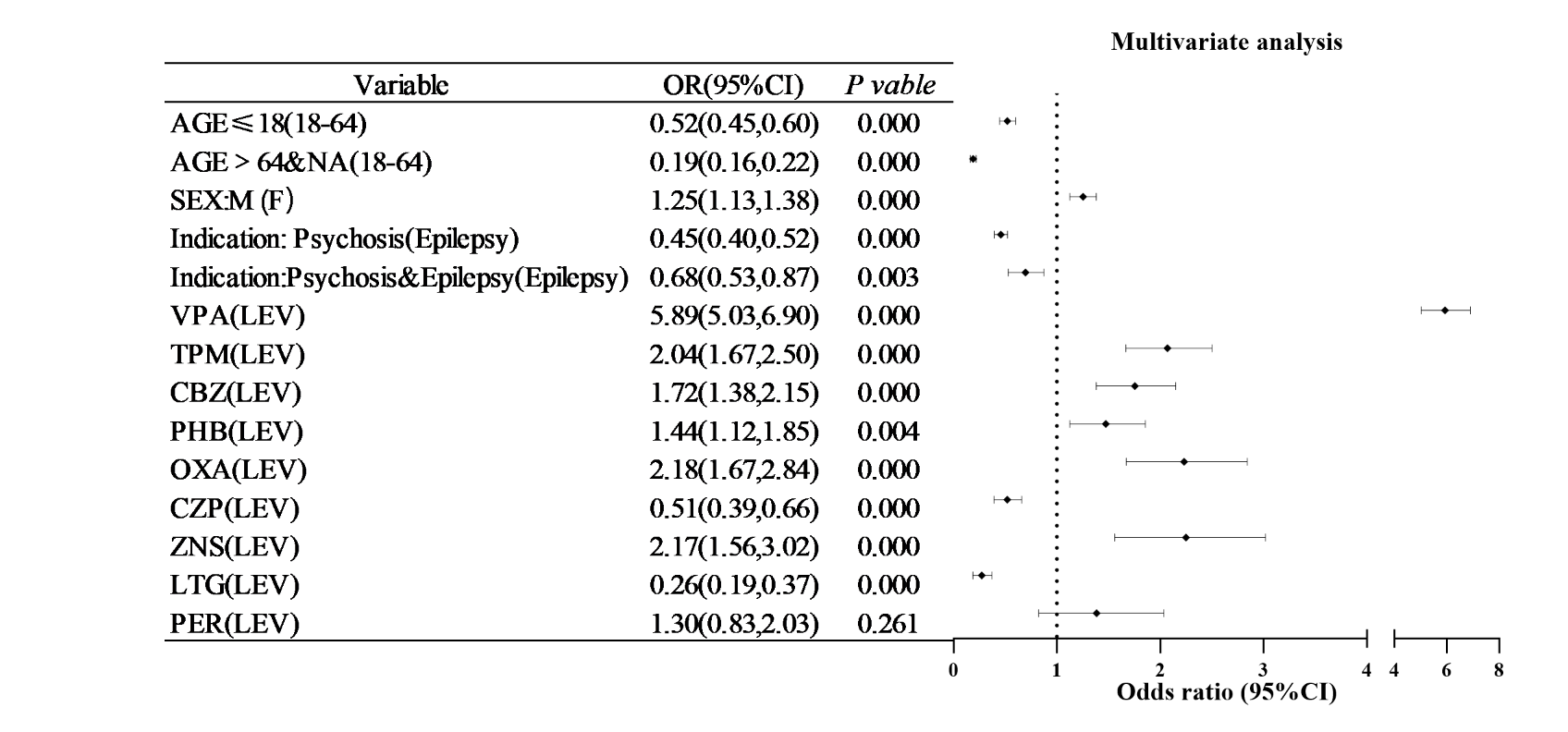


Sodium valproate (VPA); Topiramate (TPM); Levetiracetam (LEV); Phenytoin (PHT); Carbamazepine (CBZ); Oxcarbazepine (OXA); Perampanel (PER); Zornisamide (ZNS); Clonazepam (CZP); Lamotrigine (LTG);

## Supplementary Tables

Table1 Articles included in the epilepsy and psychiatric category of PT words

|  | PT |
| --- | --- |
| Epilepsy | Foetal anticonvulsant syndrome, Baltic myoclonic epilepsy, Myoclonic epilepsy and ragged-red fibres, Epilepsy congenital, Epilepsy of infancy with migrating focal seizures, Epilepsy with myoclonic-atonic seizures, Lafora's myoclonic epilepsy, Severe myoclonic epilepsy of infancy, Sudden unexplained death in epilepsy, Juvenile absence epilepsy, Petit mal epilepsy, Hypocalcaemic seizure, Seizure anoxic, Post stroke epilepsy, Post stroke seizure, Benign rolandic epilepsy, Early infantile epileptic encephalopathy with burst-suppression, Epileptic encephalopathy, Generalised tonic-clonic seizure, Hyperglycaemic seizure, Hypoglycaemic seizure, Acute encephalitis with refractory, repetitive partial seizures, Febrile infection-related epilepsy syndrome, Neonatal epileptic seizure, Neonatal seizure, Temporal lobe epilepsy, Autonomic seizure, Epilepsia partialis continua, Parietal lobe epilepsy, Acquired epileptic aphasia, Alcoholic seizure, Atypical benign partial epilepsy, Change in seizure presentation, Epilepsy, Epileptic aura, Frontal lobe epilepsy, Gelastic seizure, Hemiconvulsion-hemiplegia-epilepsy syndrome, Hyponatraemic seizure, Idiopathic generalised epilepsy, Idiopathic partial epilepsy, Juvenile myoclonic epilepsy, Migraine-triggered seizure, Myoclonic epilepsy, Partial seizures, Partial seizures with secondary generalisation, Post-traumatic epilepsy, Psychogenic seizure, Seizure cluster, Seizure like phenomena, Status epilepticus, Epilepsy surgery, Benign familial neonatal convulsions, Anticonvulsant drug level, Anticonvulsant drug level abnormal, Anticonvulsant drug level above therapeutic, Anticonvulsant drug level below therapeutic, Anticonvulsant drug level decreased, Anticonvulsant drug level increased, Anticonvulsant drug level therapeutic, Febrile convulsion, Clonic convulsion, Convulsion in childhood, Convulsions local, Convulsive threshold lowered, Drug withdrawal convulsions, Hemiconvulsion-hemiplegia-epilepsy syndrome, Photosensitive seizure, Seizure, Tonic convulsion, Seizure prophylaxis |
| Psychosis | Aberrant motor behaviour, Abnormal behaviour, Behaviour disorder, Behaviour disorder due to a general medical condition, Breath holding, Regressive behaviour, Scatolia, Sexually inappropriate behaviour, Staring, Purging, Self-induced vomiting, Abnormal dreams, Abnormal sleep-related event, Confusional arousal, Loss of dreaming, Sleep inertia, Sleep sex, Sleep talking, Sleep terror, Sleep-related eating disorder, Somnambulism, Adjustment disorder, Adjustment disorder with anxiety, Adjustment disorder with depressed mood, Adjustment disorder with disturbance of conduct, Adjustment disorder with mixed anxiety and depressed mood, Adjustment disorder with mixed disturbance of emotion and conduct, Cabin fever, Grief reaction, Persistent complex bereavement disorder, Post-sterilisation regret, Affect lability, Affective ambivalence, Blunted affect, Constricted affect, Flat affect, Inappropriate affect, Korsakoff's syndrome, Post-traumatic amnestic disorder, Paramnesia, Anorexia and bulimia syndrome, Anorexia nervosa, Bulimia nervosa, Diabulimia, Hypersomnia-bulimia syndrome, Anxiety disorder, Anxiety disorder due to a general medical condition, Generalised anxiety disorder, Neurosis, Separation anxiety disorder, Activation syndrome, Agitation, Anticipatory anxiety, Anxiety, Immunisation anxiety related reaction, Nervousness, Procedural anxiety, Stress, Tension, Eating disorder, Listless, Attention deficit hyperactivity disorder, Atypical attention deficit syndrome, Disruptive mood dysregulation disorder, Oppositional defiant disorder, Cardiovascular somatic symptom disorder, Aggression, Antisocial behaviour, Asocial behaviour, Attention-seeking behaviour, Aversion, Belligerence, Defiant behaviour, Disinhibition, Disturbance in social behaviour, Egocentrism, Grandiosity, Homicidal ideation, Hostility, Impatience, Indifference, Inferiority complex, Negativism, Overconfidence, Paranoia, Personality change, Pseudologia, Self-consciousness, Self-destructive behaviour, Social avoidant behaviour, Soliloquy, Stubbornness, Suggestibility, Suspiciousness, Violence-related symptom, Bipolar disorder, Bipolar I disorder, Bipolar II disorder, Cyclothymic disorder, Enuresis, Paruresis, Psychogenic dysuria, Breathing-related sleep disorder, Psychogenic respiratory distress, Brief psychotic disorder with marked stressors, Brief psychotic disorder without marked stressors, Brief psychotic disorder, with postpartum onset, Transient psychosis, Post stroke depression, Change in sustained attention, Daydreaming, Distractibility, Executive dysfunction, Mental fatigue, Colitis psychogenic, Communication disorder, Mutism, Speech sound disorder, Confusional state, Disorientation, Autoscopy, Habit cough, Assisted suicide, Completed suicide, Suspected suicide, Catatonia, Malignant catatonia, Withdrawal catatonia, Delirium, Delirium febrile, Delirium tremens, Intensive care unit delirium, Alice in wonderland syndrome, Delusional disorder, erotomanic type, Delusional disorder, grandiose type, Delusional disorder, jealous type, Delusional disorder, mixed type, Delusional disorder, persecutory type, Delusional disorder, somatic type, Delusional disorder, unspecified type, Cotard's syndrome, Delusion, Delusion of grandeur, Delusion of parasitosis, Delusion of reference, Delusion of replacement, Delusion of theft, Depressive delusion, Erotomanic delusion, Jealous delusion, Mixed delusion, Persecutory delusion, Somatic delusion, Thought broadcasting, Thought insertion, Thought withdrawal, Pseudodementia, Agitated depression, Childhood depression, Depression, Depression suicidal, Major depression, Menopausal depression, Mixed anxiety and depressive disorder, Perinatal depression, Persistent depressive disorder, Postictal depression, Cutaneous somatic symptom disorder, Autism spectrum disorder, Broad autism phenotype, Neurodevelopmental disorder, Depersonalisation/derealisation disorder, Dissociation, Dissociative amnesia, Dissociative disorder, Dissociative identity disorder, Near death experience, Psychogenic pseudosyncope, Sopor, Behavioural insomnia of childhood, Hyposomnia, Initial insomnia, Insomnia, Middle insomnia, Terminal insomnia, Posturing, Psychogenic movement disorder, Stereotypy, Dyssomnia, Paradoxical insomnia, Poor quality sleep, Psychophysiologic insomnia, Phonophobia, Binge eating, Merycism, Pica, Selective eating disorder, Alexithymia, Anger, Discouragement, Dysphoria, Emotional disorder, Emotional distress, Emotional poverty, Euphoric mood, Frustration tolerance decreased, Irritability, Mood altered, Morose, Neuroleptic-induced deficit syndrome, Sibling rivalry disorder, Premature ejaculation, Psychogenic erectile dysfunction, Compensation neurosis, Factitious disorder, Munchausen's syndrome, Acrophobia, Aerophobia, Agoraphobia, Aichmophobia, Algophobia, Animal phobia, Astraphobia, Autophobia, Claustrophobia, Emetophobia, Fear, Fear of animals, Fear of closed spaces, Fear of crowded places, Fear of death, Fear of disease, Fear of eating, Fear of falling, Fear of injection, Fear of open spaces, Fear of pregnancy, Fear of surgery, Fear of weight gain, Fear-related avoidance of activities, Frigophobia, Glossophobia, Haemophobia, Haphephobia, Hydrophobia, Mysophobia, Noctiphobia, Nocturnal fear, Nosocomephobia, Nosophobia, Ochlophobia, Osmophobia, Performance fear, Phagophobia, Pharmacophobia, Phobia, Phobia of driving, Phobia of exams, Phobic avoidance, Photaugiaphobia, Sitophobia, Social anxiety disorder, Social fear, Somniphobia, Thanatophobia, Thermophobia, Alcoholic hangover, Mood swings, Polydipsia psychogenic, Drug dependence, antepartum, Drug dependence, postpartum, Drug use disorder, antepartum, Bruxism, Encopresis, Gastrointestinal somatic symptom disorder, Somatoform genitourinary disorder, Hallucination, Hallucination, auditory, Hallucination, gustatory, Hallucination, olfactory, Hallucination, synaesthetic, Hallucination, tactile, Hallucination, visual, Hallucinations, mixed, Paroxysmal perceptual alteration, Somatic hallucination, Clinomania, Impulse-control disorder, Impulsive behaviour, Intermittent explosive disorder, Kleptomania, Necromania, Onychophagia, Poriomania, Pyromania, Restlessness, Disinhibited social engagement disorder of childhood, Emotional disorder of childhood, Neurotic disorder of childhood, Personality disorder of childhood, Reactive attachment disorder of infancy or early childhood, School refusal, Social (pragmatic) communication disorder, Paediatric autoimmune neuropsychiatric disorders associated with streptococcal infection, Learning disability, Learning disorder, Reading disorder, Mental disorder due to a general medical condition, Neuropsychiatric symptoms, Neuropsychiatric syndrome, Organic brain syndrome, Personality change due to a general medical condition, Tuberous sclerosis complex associated neuropsychiatric disease, Dyslogia, Mental disorder, Mental status changes, Psychological factor affecting medical condition, Bradyphrenia, Tachyphrenia, Anhedonia, Decreased interest, Depressed mood, Depressive symptom, Feeling guilty, Feeling of despair, Feelings of worthlessness, Negative thoughts, Psychomotor retardation, Sense of a foreshortened future, Tearfulness, Hypomania, Mania, Manic symptom, Affective disorder, Apathy, Boredom, Laziness, Mood disorder due to a general medical condition, Seasonal affective disorder, Substance-induced mood disorder, Torticollis psychogenic, Nail picking, Hypnagogic hallucination, Hypnopompic hallucination, Sleep attacks, Vomiting psychogenic, Neonatal complications of substance abuse, Abulia, Chronic tic disorder, Complex tic, Decreased eye contact, Neurologic somatic symptom disorder, Provisional tic disorder, Secondary tic, Thinking abnormal, Tic, Intentional self-injury, Body dysmorphic disorder, Compulsions, Compulsive handwashing, Compulsive hoarding, Compulsive shopping, Obsessive need for symmetry, Obsessive rumination, Obsessive thoughts, Obsessive-compulsive disorder, Obsessive-compulsive symptom, Olfactory reference syndrome, Trichotemnomania, Compulsive cheek biting, Compulsive lip biting, Anorgasmia, Kinesiophobia, Limited symptom panic attack, Panic attack, Panic disorder, Panic reaction, Erotophonophilia, Exhibitionism, Fetishism, Frotteurism, Masochism, Paedophilia, Paraphilia, Sadism, Transvestism, Voyeurism, Exploding head syndrome, Nightmare, Parasomnia, Rapid eye movement sleep behaviour disorder, Rapid eye movements sleep abnormal, Dopamine dysregulation syndrome, Parkinson's disease psychosis, Automatism epileptic, Epileptic psychosis, Deja vu, Delusional perception, Derealisation, Flashback, Illusion, Imperception, Jamais vu, Pseudohallucination, Time perception altered, Personality disorder, Pithiatism, Self esteem decreased, Self esteem inflated, Avoidant personality disorder, Dependent personality disorder, Obsessive-compulsive personality disorder, Antisocial personality disorder, Borderline personality disorder, Histrionic personality disorder, Narcissistic personality disorder, Psychopathic personality, Paranoid personality disorder, Schizoid personality disorder, Schizotypal personality disorder, Trichotillomania, Alcoholic psychosis, Substance-induced psychotic disorder, Drug use disorder, postpartum, Postpartum anxiety, Postpartum neurosis, Postpartum stress disorder, Helplessness, Hypervigilance, Impostor phenomenon, Neglect of personal appearance, Procrastination, Psychiatric decompensation, Psychiatric symptom, Psychological trauma, Stockholm syndrome, Trance, Acute psychosis, Childhood psychosis, Hysterical psychosis, Postictal psychosis, Psychotic behaviour, Psychotic disorder, Psychotic disorder due to a general medical condition, Psychotic symptom, Reactive psychosis, Rebound psychosis, Senile psychosis, Shared psychotic disorder, Schizoaffective disorder, Schizoaffective disorder bipolar type, Schizoaffective disorder depressive type, Schizophreniform disorder, Negative symptoms in schizophrenia, Schizophrenia, Chronic idiopathic pain syndrome, Phantom vibration syndrome, Gender dysphoria, Psychosexual disorder, Disturbance in sexual arousal, Sexual inhibition, Compulsive sexual behaviour, Excessive masturbation, Excessive sexual fantasies, Hypersexuality, Loss of libido, Sexual aversion disorder, Female orgasmic disorder, Genito-pelvic pain/penetration disorder, Libido decreased, Libido disorder, Libido increased, Male orgasmic disorder, Orgasm abnormal, Orgasmic sensation decreased, Dermatillomania, Dermatophagia, Sleep disorder, Sleep disorder due to a general medical condition, Sleep disorder due to general medical condition, hypersomnia type, Sleep disorder due to general medical condition, insomnia type, Sleep disorder due to general medical condition, mixed type, Sleep disorder due to general medical condition, parasomnia type, Conversion disorder, Illness anxiety disorder, Pseudoneurologic symptom, Somatic symptom disorder, Aphonia psychogenic, Coprolalia, Disorganised speech, Dysphemia, Dysphonia psychogenic, Echolalia, Logorrhoea, Neologism, Pedantic speech, Poverty of speech, Selective mutism, Taciturnity, Verbigeration, Clang associations, Lack of spontaneous speech, Pressure of speech, Automatism, Automatism, command, Echopraxia, Finger licking, Head banging, Waxy flexibility, Acute stress disorder, Anniversary reaction, Burnout syndrome, Catastrophic reaction, Hyperarousal, Post-traumatic stress disorder, Ulysses syndrome, Alcohol abuse, Alcohol problem, Alcohol use disorder, Alcoholism, Behavioural addiction, Binge drinking, Caffeine dependence, Dependence, Drug abuse, Drug dependence, Drug use disorder, Gambling disorder, Gaming disorder, Nicotine dependence, Substance abuse, Substance dependence, Substance use disorder, Tobacco abuse, Self-injurious ideation, Suicidal behaviour, Suicidal ideation, Suicide attempt, Suicide threat, Suspected suicide attempt, Circumstantiality, Confabulation, Derailment, Flight of ideas, Ideas of reference, Illogical thinking, Impaired reasoning, Intellectualisation, Intrusive thoughts, Loose associations, Magical thinking, Morbid thoughts, Paralogism, Pathological doubt, Perseveration, Poverty of thought content, Tangentiality, Thought blocking, Psychogenic tremor, Psychogenic blindness, Psychogenic visual disorder, Alcohol withdrawal syndrome, Tobacco withdrawal symptoms |

Table2 Scientific terms

| **Abbreviations** | **Full name** |
| --- | --- |
| HE | Hyperammonemic encephalopathy |
| OR | Odds ratio |
| ROR | Report odds ratio |
| PRR | Proportional reporting rate |
| CI | Confidence interval |
| DDIs | Drug-drug interactions |
| AED | Antiepileptic drug |
| APD | Antipsychotic drug |
| VPA | Sodium valproate |
| TPM | Topiramate |
| LEV | Levetiracetam |
| PHT | Phenytoin |
| CBZ | Carbamazepine |
| OXA | Oxcarbazepine |
| PER | Perampanel |
| ZNS | Zornisamide |
| CZP | Clonazepam |
| LTG | Lamotrigine |
| QTP | Quetiapine |
| OLZ | Olanzapine |
| DE | Death |
| LT | Life-threatening |
| HO | Hospitalization-initial or prolonged |
| DS | Disability |
| CA | Congenital anomaly |
| RI | Required intervention |
| OT | Other serious events |

Table3

**Four****-fold table of AEs**

| **Drugs** | **Target AEs** | **Other AEs** | **Total** |
| --- | --- | --- | --- |
| **Target drugs** | a | b | a+ b |
| **Other drugs** | c | d | c+ d |
| **Total** | a+ c | b+ d | a+ b+ c+ d |

Report odds ratio (ROR); adverse event (AE);

ROR=(a/c)/(b/d); ROR 95%*CI*= exp (ln (ROR) ±1.96 $\sqrt{\frac{1}{a}+\frac{1}{b}+\frac{1}{c}+\frac{1}{d}}$)

Table4

**The 4 × 2 contingency table for signal detection.**

|  | **Target AE** | **All other AEs** | **Total** |
| --- | --- | --- | --- |
| **drug D_1_ and drug D_2_** | n_111_ | n_110_ | n_11+_ |
| **Only drug D_1_** | n_101_ | n_100_ | n_10+_ |
| **Only drug D2** | n_011_ | n_010_ | n_01+_ |
| **Neither drug D_1_ and drug D_2_** | n_001_ | n_000_ | n_00+_ |
| **Total** | n_++1_ | n_++0_ | n_+++_ |

Table5

**The 2 × 2 contingency table for signal detection.**

|  | **drug D_2_** | **Not drug D_2_** |
| --- | --- | --- |
| **drug D_1_** | *P_11_*  (=n_111_/n_11+_) | *P_10_*  (=n_101_/n_10+_) |
| **Not drug D_1_** | *P_01_*  (=n_011_/n_01+_) | *P_00_*  (=n_001_/n_00+_) |

Additive Model: *P_11_* – *P_00_* = (*P_11_* – *P_00_*) + (*P_11_* – *P_00_*)

Table6 Demographic distribution of the 10 AEDs (**VPA not excluded)**

|  |  | VPA | LEV | TPM | CBZ | PHT | OXA | CLZ | ZNS | LTG | PER | Total |
| --- | --- | --- | --- | --- | --- | --- | --- | --- | --- | --- | --- | --- |
| Total reports | | 639 | 144 | 136 | 82 | 60 | 62 | 49 | 43 | 28 | 21 | 1264 |
| Sex distribution | | |  |  |  |  |  |  |  |  |  |  |
|  | Female | 314(49.14%) | 75(52.08%) | 67(49.26%) | 45(54.88%) | 23(38.33%) | 30(48.39%) | 30(61.22%) | 17(39.53%) | 7(25.00%) | 9(42.86%) | 617(48.81%) |
|  | Male | 275(43.04%) | 58(40.28%) | 51(37.50%) | 34(41.46%) | 37(61.67%) | 26(41.94%) | 14(28.57%) | 22(51.16%) | 17(60.71%) | 11(52.38%) | 545(43.12%) |
|  | NA | 50(7.82%) | 11(7.64%) | 18(13.24%) | 3(3.66%) |  | 6(9.68%) | 5(10.20%) | 4(9.30%) | 4(14.29%) | 1(4.76%) | 102(8.07%) |
| Age distribution | | |  |  |  |  |  |  |  |  |  |  |
|  | ＜18 | 88(13.77%) | 18(12.50%) | 18(13.24%) | 9(10.98%) | 14(23.33%) | 24(38.71%) | 6(12.24%) | 4(9.30%) | 2(7.14%) | 5(23.81%) | 188(14.87%) |
|  | 18-64 | 455(71.21%) | 110(76.39%) | 94(69.12%) | 67(81.71%) | 42(70.00%) | 29(46.77%) | 33(67.35%) | 31(72.09%) | 18(64.29%) | 15(71.43%) | 894(70.73%) |
|  | ＞64 | 42(6.57%) | 5(3.47%) | 2(1.47%) | 3(3.66%) | 4(6.67%) |  | 1(2.04%) |  | 4(14.29%) |  | 61(4.83%) |
|  | NA | 54(8.45%) | 11(7.64%) | 22(16.18%) | 3(3.66%) |  | 9(14.52%) | 9(18.37%) | 8(18.60%) | 4(14.29%) | 1(4.76%) | 121(9.57%) |
| Severity | |  |  |  |  |  |  |  |  |  |  |  |
|  | DE | 42(6.57%) | 7(4.86%) |  | 7(8.54%) | 3(5.00%) |  |  |  | 5(17.86%) |  | 64(5.06%) |
|  | LT | 99(15.49%) | 27(18.75%) | 7(5.15%) | 12(14.63%) | 6(10.00%) | 13(20.97%) | 8(16.33%) | 12(27.91%) | 2(7.14%) | 7(33.33%) | 193(15.27%) |
|  | HO | 342(53.52%) | 75(52.08%) | 96(70.59%) | 49(59.76%) | 36(60.00%) | 41(66.13%) | 23(46.94%) | 25(58.14%) | 19(67.86%) | 14(66.67%) | 720(56.96%) |
|  | DS | 1(0.16%) |  |  |  |  |  |  |  |  |  | 1(0.08%) |
|  | OT | 153(23.94%) | 35(24.31%) | 32(23.53%) | 14(17.07%) | 14(23.33%) | 8(12.90%) | 18(36.73%) | 6(13.95%) | 2(7.14%) |  | 282(22.31%) |
|  | NA | 2(0.31%) |  | 1(0.74%) |  | 1(1.67%) |  |  |  |  |  | 4(0.32%) |
| Geographcial distribution | | |  |  |  |  |  |  |  |  |  |  |
|  | America | 200(31.30%) | 50(34.72%) | 70(51.47%) | 34(41.46%) | 25(41.67%) | 15(24.19%) | 13(26.53%) | 22(51.16%) | 10(35.71%) | 11(52.38%) | 450(35.60%) |
|  | Europe | 180(28.17%) | 50(34.72%) | 41(30.15%) | 11(13.41%) | 15(25.00%) | 26(41.94%) | 14(28.57%) | 3(6.98%) | 13(46.43%) | 10(47.62%) | 363(28.72%) |
|  | Asia | 160(25.04%) | 30(20.83%) | 6(4.41%) | 30(36.59%) | 9(15.00%) | 14(22.58%) | 18(36.73%) | 13(30.23%) | 1(3.57%) |  | 281(22.23%) |
|  | Oceania | 22(3.44%) | 1(0.69%) | 8(5.88%) |  | 1(1.67%) |  |  |  |  |  | 32(2.53%) |
|  | Africa |  |  |  |  |  |  |  |  |  |  |  |
|  | NA | 77(12.05%) | 13(9.03%) | 11(8.09%) | 7(8.54%) | 10(16.67%) | 7(11.29%) | 4(8.16%) | 5(11.63%) | 4(14.29%) |  | 138(10.92%) |

Sodium valproate (VPA); Topiramate (TPM); Levetiracetam (LEV); Phenytoin (PHT); Carbamazepine (CBZ); Oxcarbazepine (OXA); Perampanel (PER); Zornisamide (ZNS); Clonazepam (CZP); Lamotrigine (LTG); Death (DE); Life-threatening (LT); Hospitalization-initial or prolonged (HO); Disability (DS); Congenital anomaly (CA); Required intervention (RI); Other serious events (OT);

Table7 Demographic distribution of the 10 AEDs

|  |  | VPA | LEV | TPM | CBZ | PHT | OXA | CLZ | ZNS | LTG | PER | Total |
| --- | --- | --- | --- | --- | --- | --- | --- | --- | --- | --- | --- | --- |
| Total reports | |  | 639 | 16 | 46 | 30 | 30 | 14 | 6 | 1 | 11 | 16 |
| Sex distribution | | |  |  |  |  |  |  |  |  |  |  |
|  | Female | 314(49.14%) | 8(50.00%) | 13(28.26%) | 11(36.67%) | 10(33.33%) | 4(28.57%) | 2(33.33%) |  | 2(18.18%) | 4(25.00%) | 368(45.49%) |
|  | Male | 275(43.04%) | 6(37.50%) | 28(60.87%) | 19(63.33%) | 20(66.67%) | 8(57.14%) | 3(50.00%) | 1(100.00%) | 7(63.64%) | 11(68.75%) | 378(46.72%) |
|  | NA | 50(7.82%) | 2(12.50%) | 5(10.87%) |  |  | 2(14.29%) | 1(16.67%) |  | 2(18.18%) | 1(6.25%) | 63(7.79%) |
| Age distribution | | |  |  |  |  |  |  |  |  |  |  |
|  | ＜18 | 88(13.77%) |  | 4(8.70%) | 1(3.33%) | 4(13.33%) | 6(42.86%) | 2(33.33%) |  | 2(18.18%) | 5(31.25%) | 112(13.84%) |
|  | 18-64 | 455(71.21%) | 12(75.00%) | 38(82.61%) | 27(90.00%) | 25(83.33%) | 7(50.00%) | 3(50.00%) | 1(100.00%) | 5(45.45%) | 10(62.50%) | 583(72.06%) |
|  | ＞64 | 42(6.57%) | 2(12.50%) |  | 2(6.67%) | 1(3.33%) |  |  |  | 2(18.18%) |  | 49(6.06%) |
|  | NA | 54(8.45%) | 2(12.50%) | 4(8.70%) |  |  | 1(7.14%) | 1(16.67%) |  | 2(18.18%) | 1(6.25%) | 65(8.03%) |
| Severity | |  |  |  |  |  |  |  |  |  |  |  |
|  | DE | 42(6.57%) | 1(6.25%) |  | 3(10.00%) | 1(3.33%) |  |  |  | 4(36.36%) |  | 51(6.30%) |
|  | LT | 99(15.49%) | 1(6.25%) | 2(4.35%) | 1(3.33%) | 2(6.67%) | 6(42.86%) | 1(16.67%) |  | 1(9.09%) | 5(31.25%) | 118(14.59%) |
|  | HO | 342(53.52%) | 10(62.50%) | 35(76.09%) | 22(73.33%) | 21(70.00%) | 7(50.00%) | 4(66.67%) | 1(100.00%) | 5(45.45%) | 11(68.75%) | 458(56.61%) |
|  | DS | 1(0.16%) |  |  |  |  |  |  |  |  |  | 1(0.12%) |
|  | OT | 153(23.94%) | 4(25.00%) | 9(19.57%) | 4(13.33%) | 6(20.00%) | 1(7.14%) | 1(16.67%) |  | 1(9.09%) |  | 179(22.13%) |
|  | NA | 2(0.31%) |  |  |  |  |  |  |  |  |  | 2(0.25%) |
| Geographcial distribution | | |  |  |  |  |  |  |  |  |  |  |
|  | | |  |  |  |  |  |  |  |  |  |  |
|  | America | 200(31.30%) | 8(50.00%) | 29(63.04%) | 25(83.33%) | 17(56.67%) | 1(7.14%) | 1(16.67%) | 1(100.00%) | 6(54.55%) | 11(68.75%) | 299(36.96%) |
|  | Europe | 180(28.17%) | 6(37.50%) | 9(19.57%) | 2(6.67%) | 3(10.00%) | 11(78.57%) | 1(16.67%) |  | 3(27.27%) | 5(31.25%) | 220(27.19%) |
|  | Asia | 160(25.04%) | 1(6.25%) | 2(4.35%) | 3(10.00%) | 8(26.67%) | 1(7.14%) | 2(33.33%) |  | 0.00% | 0.00% | 177(21.88%) |
|  | Oceania | 22(3.44%) |  | 1(2.17%) |  |  |  |  |  |  |  | 23(2.84%) |
|  | Africa |  |  |  |  |  |  |  |  |  |  |  |
|  | NA | 77(12.05%) | 1(6.25%) | 5(10.87%) |  | 2(6.67%) | 1(7.14%) | 2(33.33%) |  | 2(18.18%) |  | 90(11.12%) |

Sodium valproate (VPA); Topiramate (TPM); Levetiracetam (LEV); Phenytoin (PHT); Carbamazepine (CBZ); Oxcarbazepine (OXA); Perampanel (PER); Zornisamide (ZNS); Clonazepam (CZP); Lamotrigine (LTG); Death (DE); Life-threatening (LT); Hospitalization-initial or prolonged (HO); Disability (DS); Congenital anomaly (CA); Required intervention (RI); Other serious events (OT);

Table8 Demographic distribution of hyperammonemic encephalopathy caused by sodium valproate combined with antiepileptic drugs (topiramate or levetiracetam)

|  |  | VPA | TPM+VPA | LEV+VPA | TPM+LEV+VPA | Total |
| --- | --- | --- | --- | --- | --- | --- |
| Total reports | | 457 | 56 | 93 | 35 | 641 |
| Sex distribution | |  |  |  |  |  |
|  | Female | 218(47.70%) | 31(55.36%) | 43(46.24%) | 24(68.57%) | 316(49.30%) |
|  | Male | 207(45.30%) | 16(28.57%) | 45(48.39%) | 7(20.00%) | 275(42.90%) |
|  | NA | 32(7.00%) | 9(16.07%) | 5(5.38%) | 4(11.43%) | 50(7.80%) |
| Age distribution | |  |  |  |  |  |
|  | ＜18 | 59(12.91%) | 12(21.43%) | 15(16.13%) | 3(8.57%) | 89(13.88%) |
|  | 18-64 | 328(71.77%) | 30(53.57%) | 72(77.42%) | 26(74.29%) | 456(71.14%) |
|  | ＞64&NA | 70(15.32%) | 14(25.00%) | 6(6.45%) | 6(17.14%) | 96(14.98%) |
| Severity | |  |  |  |  |  |
|  | DE | 36(7.88%) |  | 6(6.45%) |  | 42(6.55%) |
|  | LT | 71(15.54%) | 4(7.14%) | 23(24.73%) | 3(8.57%) | 101(15.76%) |
|  | HO | 242(52.95%) | 35(62.50%) | 40(43.01%) |  | 342(53.35%) |
|  | DS | 1(0.22%) |  |  |  | 1(0.0.16%) |
|  | OT | 106(23.19%) | 16(28.57%) | 24(25.81%) | 32(91.43%) | 153(23.87%) |
|  | NA | 1(0.22%) | 1(1.79%) |  |  |  |

Sodium valproate (VPA); Topiramate (TPM); Levetiracetam (LEV); Death (DE); Life-threatening (LT); Hospitalization-initial or prolonged (HO); Disability (DS); Congenital anomaly (CA); Required intervention (RI); Other serious events (OT);

Table9 Demographic distribution of hyperammonemic encephalopathy caused by sodium valproate combined with antiepileptic drugs (topiramate or levetiracetam) **(epilepsy patients)**

|  |  | VPA | TPM+VPA | LEV+VPA | TPM+LEV+VPA | Total |
| --- | --- | --- | --- | --- | --- | --- |
| Total reports | | 97 | 33 | 71 | 24 | 225 |
| Sex distribution | |  |  |  |  |  |
|  | Female | 37(38.14%) | 18(54.55%) | 35(49.30%) | 16(66.67%) | 106(47.11%) |
|  | Male | 54(55.67%) | 8(24.24%) | 33(46.48%) | 5(20.83%) | 100(44.44%) |
|  | NA | 6(6.19%) | 7(21.21%) | 3(4.23%) | 3(12.50%) | 19(8.44%) |
| Age distribution | |  |  |  |  |  |
|  | ＜18 | 19(19.59%) | 9(27.27%) | 13(18.31%) | 3(12.50%) | 44(19.56%) |
|  | 18-64 | 56(57.73%) | 16(48.48%) | 55(77.46%) | 17(70.83%) | 144(64.00%) |
|  | ＞64&NA | 22(22.68%) | 8(24.24%) | 3(4.23%) | 4(16.67%) | 37(16.44%) |
|  | |  |  |  |  |  |
| Severity |  |  |  |  |  |  |
|  | DE | 15(15.46%) |  | 4(5.63%) |  | 19(8.44%) |
|  | LT | 11(11.34%) | 2(6.06%) | 17(23.94%) | 1(4.17%) | 31(13.78%) |
|  | HO | 44(45.36%) | 22(66.67%) | 28(39.44%) | 18(75.00%) | 112(49.78%) |
|  | OT | 106(23.19%) | 16(28.57%) | 24(25.81%) | 32(91.43%) | 153(23.87%) |
|  | NA | 1(0.22%) | 1(1.79%) |  |  |  |

Sodium valproate (VPA); Topiramate (TPM); Levetiracetam (LEV); Death (DE); Life-threatening (LT); Hospitalization-initial or prolonged (HO); Disability (DS); Congenital anomaly (CA); Required intervention (RI); Other serious events (OT);

Table10 Demographic distribution of hyperammonemic encephalopathy caused by sodium valproate combined with antipsychotic drugs (quetiapine or olanzapine)

|  |  | VPA | QTP+VPA | OLZ+VPA | QTP+OLZ+VPA | Total |
| --- | --- | --- | --- | --- | --- | --- |
| Total reports | | 457 | 460 | 100 | 61 | 18 |
| Sex distribution | |  |  |  |  |  |
|  | Female | 207(45.00%) | 65(65.00%) | 27(44.26%) | 15(83.33%) | 314(49.14%) |
|  | Male | 215(46.74%) | 30(30.00%) | 29(47.54%) | 1(5.56%) | 275(43.04%) |
|  | NA | 38(8.26%) | 5(5.00%) | 5(8.20%) | 2(11.11%) | 50(7.82%) |
| Age distribution | |  |  |  |  |  |
|  | ＜18 | 93(20.22%) | 1(10.00%) | 4(6.56%) |  | 98(15.34%) |
|  | 18-64 | 308(66.96%) | 81(81.00%) | 50(81.97%) | 16(88.89%) | 455(71.21%) |
|  | ＞64&NA | 59(12.83%) | 18(18.00%) | 7(11.48%) | 2(11.11%) | 86(13.46%) |
| Severity | |  |  |  |  |  |
|  | DE | 37(8.04%) | 1(10.00%) |  |  | 38(5.95%) |
|  | LT | 46(10.00%) | 18(18.00%) | 18(29.51%) | 9(50.00%) | 91(14.24%) |
|  | HO | 255(55.43%) | 58(58.00%) | 33(54.10%) | 8(44.44%) | 354(55.40%) |
|  | DS | 1(0.22%) |  |  |  | 1(0.16%) |
|  | OT | 119(25.87%) | 23(23.00%) | 10(16.39%) | 1(5.56%) | 153(23.94%) |
|  | NA | 2(0.43%) |  |  |  | 2(0.31%) |

Sodium valproate (VPA); Quetiapine (QTP); Olanzapine (OLZ); Death (DE); Life-threatening (LT); Hospitalization-initial or prolonged (HO); Disability (DS); Congenital anomaly (CA); Required intervention (RI); Other serious events (OT);

Table11 Demographic distribution of hyperammonemic encephalopathy caused by sodium valproate combined with antipsychotic drugs (quetiapine or olanzapine) **(Psychosis patients)**

|  |  | VPA | QTP+VPA | OLZ+VPA | QTP+OLZ+VPA | Total |
| --- | --- | --- | --- | --- | --- | --- |
| Total reports | | 128 | 63 | 51 | 17 | 259 |
| Sex distribution | |  |  |  |  |  |
|  | Female | 53(41.41%) | 44(69.84%) | 21(41.18%) | 15(88.24%) | 133(51.35%) |
|  | Male | 61(46.66%) | 15(10.143.81%) | 25(49.02%) |  | 101(39.00%) |
|  | NA | 14(10.94%) | 4(6.35%) | 5(9.80%) | 2(11.76%) | 25(9.65%) |
| Age distribution | |  |  |  |  |  |
|  | ＜18 | 14(10.94%) |  | 2(3.92%) |  | 16(6.18%) |
|  | 18-64 | 99(77.34%) | 48(76.19%) | 42(82.35%) | 15(88.24%) | 204(78.76%) |
|  | ＞64&NA | 15(11.72%) | 15(23.81%) | 7(13.73%) | 2(11.76%) | 39(15.06%) |
|  | |  |  |  |  |  |
| Severity |  |  |  |  |  |  |
|  | DE | 7(5.47%) | 1(1.59%) |  |  | 8(3.09%) |
|  | LT | 12(9.38%) | 13(20.63%) | 15(29.41%) | 9(52.94%) | 49(18.92%) |
|  | HO | 76(59.38%) | 39(61.90%) | 28(54.90%) | 7(41.18%) | 150(57.92%) |
|  | OT |  |  |  |  |  |
|  | NA | 33(25.78%) | 10(15.87%) | 8(15.69%) | 1(5.88%) | 52(20.08%) |

Sodium valproate (VPA); Quetiapine (QTP); Olanzapine (OLZ); Death (DE); Life-threatening (LT); Hospitalization-initial or prolonged (HO); Disability (DS); Congenital anomaly (CA); Required intervention (RI); Other serious events (OT);

Table12 Risk for hyperammonemic encephalopathy (HE) associated with 10 antiepileptic drugs (AEDs)

|  | a | ROR | ROR95%CI | PRR | PRR95%CI | χ2 | *P* |
| --- | --- | --- | --- | --- | --- | --- | --- |
| VPA | 666 | 122.14 | (110.16,135.41) | 121.88 | (109.95,135.11) | 43258.05 | 0.000 |
| LEV | 150 | 14.38 | (12.15,17.03) | 14.38 | (12.14,17.02) | 1662.52 | 0.000 |
| non-VPA | 16 | 1.61 | (0.99,2.64) | 1.61 | (0.99,2.64) | 3.10 | 0.079 |
| TPM | 149 | 16.71 | (14.1,19.8) | 16.70 | (14.10,19.79) | 1960.42 | 0.000 |
| non-VPA | 52 | 5.95 | (4.51,7.85) | 5.95 | (4.51,7.85) | 201.91 | 0.000 |
| CBZ | 88 | 14.84 | (11.96,18.41) | 14.83 | (11.96,18.4) | 1053.60 | 0.000 |
| non-VPA | 35 | 6.45 | (4.61,9.02) | 6.45 | (4.61,9.02) | 151.91 | 0.000 |
| PHB | 70 | 21.42 | (16.84,27.23) | 21.40 | (16.83,27.21) | 1276.61 | 0.000 |
| non-VPA | 35 | 12.60 | (9.01,17.62) | 12.59 | (9,17.61) | 353.27 | 0.000 |
| OXA | 66 | 25.86 | (20.2,33.1) | 25.84 | (20.19,33.07) | 1480.85 | 0.000 |
| non-VPA | 14 | 6.13 | (3.62,10.37) | 6.13 | (3.62,10.37) | 54.50 | 0.000 |
| CZP | 57 | 2.70 | (2.07,3.52) | 2.70 | (2.07,3.52) | 57.13 | 0.000 |
| non-VPA | 6 | 0.29 | (0.13,0.65) | 0.29 | (0.13,0.65) | 9.55 | 0.002 |
| ZNS | 49 | 40.32 | (30.32,53.61) | 40.27 | (30.29,53.53) | 1775.41 | 0.000 |
| non-VPA | 1 | 0.99 | (0.14,7.06) | 0.99 | (0.14,7.06) | 0.00 | 1.000 |
| LTG | 34 | 3.07 | (2.18,4.31) | 3.07 | (2.18,4.31) | 44.24 | 0.000 |
| non-VPA | 11 | 1.11 | (0.61,2.01) | 1.11 | (0.61,2.01) | 0.04 | 0.848 |
| PER | 26 | 45.54 | (30.89,67.14) | 45.48 | (30.87,67.01) | 1067.60 | 0.000 |
| non-VPA | 21 | 52.62 | (34.19,80.99) | 52.54 | (34.16,80.8) | 996.17 | 0.000 |

Sodium valproate (VPA); Topiramate (TPM); Levetiracetam (LEV); Phenytoin (PHT); Carbamazepine (CBZ); Oxcarbazepine (OXA); Perampanel (PER); Zornisamide (ZNS); Clonazepam (CZP); Lamotrigine (LTG); Report odds ratio (ROR); Proportional reporting rate (PRR); Chi-square (χ2);
